# Risk factors identification of COVID‐19 patients with chronic obstructive pulmonary disease: A retrospective study in Punjab‐Pakistan

**DOI:** 10.1002/iid3.981

**Published:** 2023-08-28

**Authors:** Muhammad Muneeb Hassan, M. H. Tahir, Muhammad Ameeq, Farrukh Jamal, John T. Mendy, Christophe Chesneau

**Affiliations:** ^1^ Department of Statistics The Islamia University of Bahawalpur Bahawalpur Punjab Pakistan; ^2^ Department of Mathematics, School of Arts and Science University of The Gambia Serrekunda Gambia; ^3^ Department of Mathematics, LMNO University of Caen‐Normandie Caen France

**Keywords:** COPD, COVID‐19, Cox proportional hazard model and negative binomial distribution, SARS‐CoV‐2

## Abstract

**Background:**

Accessibility to the immense collection of studies on noncommunicable diseases related to coronavirus disease of 2019 (COVID‐19) and severe acute respiratory syndrome coronavirus 2 (SARS‐CoV‐2) is an immediate focus of researchers. However, there is a scarcity of information about chronic obstructed pulmonary disease (COPD), which is associated with a high rate of infection in COVID‐19 patients. Moreover, by combining the effects of the SARS‐CoV‐2 on COPD patients, we may be able to overcome formidable obstacles factors, and diagnosis influencers.

**Materials and Methods:**

A retrospective study of 280 patients was conducted at DHQ Hospital Muzaffargarh in Punjab, Pakistan. Negative binomial regression describes the risk of fixed successive variables. The association is described by the Cox proportional hazard model and the model coefficient is determined through log‐likelihood observation. Patients with COPD had their survival and mortality plotted on Kaplan–Meier curves.

**Results:**

The increased risk of death in COPD patients was due to the effects of variables such as cough, lower respiratory tract infection (LRTI), tuberculosis (TB), and body‐aches being 1.369, 0.693, 0.170, and 0.217 times higher at (95% confidence interval [CI]: 0.747–1.992), (95% CI: 0.231–1.156), (95% CI: 0.008–0.332), and (95% CI: −0.07 to 0.440) while it decreased 0.396 in normal condition.

**Conclusion:**

We found that the symptoms of COPD (cough, LRTI, TB, and bodyaches) are statistically significant in patients who were most infected by SARS‐CoV‐2.

## Graphical abstract

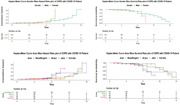

## 1 INTRODUCTION

In recent years, there have been many important changes in the medical field. Clinical and scientific professionals focused on patients with chronic obstruct pulmonary disease (COPD), who may be more likely to get infected with coronavirus disease of 2019 (COVID‐19) than other people.^1^ The severe acute respiratory syndrome corona virus‐2 (SARS‐CoV‐2)^2^ pandemic also has a higher relative risk, with over 240 million people affected and approximately 4.9 million deaths occurring due to chronic disease.

The European Respiratory Society, the Japanese Respiratory Society, and the Latin American Thoracic Association are working on reliable COPD diagnostic guidelines.^3^, ^4^ Industrialization and urban population areas were affected by air pollution during the lockdown. The controlled disease disrupts the environment by increasing illness severity, mortality rates, and risk for those in the affected environment. Under control or containment, this disease disrupts the environment's regular operations, increasing illness and the risk to that exposed.^5^ Most patients who already had diabetes, respiratory disorders, cardiovascular disease, and COPD were infected all over the world.^6^

Over 79 million cases of COVID‐19 have been confirmed worldwide since the World Health Organization (WHO) declared the disease pandemic in 2020, with 6,444,316 deaths on December 5, 2022. During COVID‐19, all over the world, 0%–10% of patient were diagnosed with COPD. The prevalence of COPD in Sub‐Saharan Africa is between 4.1% and 24.8%, despite the region's high incidence of infectious diseases like HIV and tuberculosis that serve as major risk factors for COPD.^7^ The prevalence of COPD in Europe ranges from 5.6% to 11.1%, whereas in the United States, it ranges from 2.4% to 5.4%.^8^ In COVID‐19, the WHO boosts work in noncommunicable disease (NCD) clinics around the world to help diagnose and treat the condition. Because of the COVID‐19 disease, COPD has become a top priority in the UN‐2030 Agenda for Sustainable Development and the WHO Global Action‐Plan for the Prevention and Control of Non‐communicable Diseases.^9^

The disease COPD affects not only a country's healthcare system but also the quality of life of patients and their families. In Pakistan, from January 3, 2020, to December 3, 2022, there were 1,575,486 confirmed cases of COVID‐19, with 30,635 deaths reported to the WHO,^10^ and prevalence of COPD among adults aged 40 and older was found at 2.1%.^11^

### 1.1 Consequences of the study

To confront the escalating causes of these diseases, improved policymaking and intervention, it is necessary to examine the connections between the diseases and the factors that influence them. In the present analysis, 12 independent factors (diagnosis) such as body‐aches, wheezing, headache, normal condition, shortening of breath (SOB), tuberculosis (TB), lower respiratory tract infection (LRTI), Acid Fast bacillus (AFB), right‐side chest pain, sputum, cough, and fever were selected to examine the impact of risk factors for COPD and to explore the link between different risk factors.

## 2 MATERIALS AND METHODS

### 2.1 Study population

In this study, secondary data from 280 male and female COPD patients with COVID‐19 were taken from a population of 4,348,549^12^ in District Muzaffar‐garh, Punjab, Pakistan. There were a total of four subdistricts namely Muzaffar‐garh, Jatoi, Ali‐Pur, and Kot‐Adu, each encompassing both rural and urban areas. The study's duration was from February to August of 2022, with the collection of information wrapping up in March of that year. Using a simple random sampling technique, we included COPD patients who had COVID‐19 and were admitted from January 2020 to December 2021. Spirometry, a routine breathing test, was used to identify COPD cases. Although there is currently no laboratory test that can definitively diagnose COPD, there are a number of tests that can be performed on a COPD patient to rule out other potential causes of dyspnea and to identify co‐existing conditions. Routine blood work is important to rule out anemia, as anemic patients may also present with a history of shortness of breath. Checking plasma levels of brain natriuretic peptide (BNP) or N‐terminal pro‐BNP is the gold standard for ruling out heart failure (NT‐proBNP). Positive real‐time reverse transcription polymerase chain reaction (RT‐PCR) results were obtained from laboratories at the Research Institute of Nishtar Medical College and University in Multan, both of which are recognized by the Punjab Health Council (PHC). The data and detailed information were acquired by a nurse or a consultant pulmonologist after a standard medical checkup. The patient's resource files were manually examined for the interview form. Independent factors (diagnosis) included bodyaches, wheezing, headache, normal condition, SOB, TB, LRTI, AFB, right‐side chest pain, sputum, cough, and fever,^13^, ^14^, ^15^, ^16^, ^17^, ^18^, ^19^, ^20^, ^21^, ^22^, ^23^ which were all based on lung infections. Dependent variables included gender, age, and location (distributed in subdistrict). We include only confirmed COVID‐19 patients who also had COPD and exclude all COPD patients who were not confirmed. The DHQ Hospital Muzaffar‐garh Statistical Officer in charge of the ethics committee provided a letter of clearance for the study on May 10, 2022, citing the number 1174‐77/DHQ. The SPSS‐22, Math‐Type, and R packages were used for all statistical analysis.

### 2.2 Model specification

#### 2.2.1 Negative binomial distribution

In geometric experiments, it is only interesting if the first success occurs, and we repeat the experiment even if the first success does not occur. However, in many situations, this extended to a fixed number of successes rather than a single source.^24^

#### 2.2.2 Distribution summary

*p* and *k* two‐parameter used in negative distribution.

*p* = probability of success.

*k* = number of success.

Pmf:

(1) P(X=x)=x−1k−1pkqx−k;x=k,k+1,k+2,…

where

*X* = number of trials to produce k successes.

*q* = 1 − *p*

#### 2.2.3 Negative binomial regression model

(2) $$U_{i}=\vartheta .K_{i}+\varepsilon_{i},$$

where “ϑ” is the set of regression coefficient and “$K_{i}$” is the set of independent variables describing a person “ι” which may be either “dummy variables” or regular continuous variables.^25^, ^26^, ^27^, ^28^, ^29^

#### 2.2.4 Cox proportional hazard model

It is used to evaluate the simultaneous effects of different factors on survival. It identifies specific factors of influence or events happening (e.g., disease, infection rate, etc.). A Hazard‐function is denoted by *h* (*t*_1_) which can be interpreted as a risk with respect to time *t*_1_. It can be calculated as follows^30^, ^31^:

(3) $$h\left(t\right)=h_{0}\left(t_{1}\right)\times \text{exp}(b_{a1}x_{a1}+b_{b2}x_{b2}+b_{c3}x_{c3}+\ldots +b_{\text{qp}}x_{\text{qp}}),$$

where

1. *t*_1_ represent the survival time.
2. *h*(*t*_1_) functions determine the *p* covariate ($x_{a1},x_{b2},x_{c3}\ldots .x_{\mathrm{qp}}$) with coefficient ($b_{a1},b_{b2},b_{c3}\ldots .b_{\mathrm{qp}}$) which measure the covariates in Equation (3).
  as
3. (H.R = 1): No‐effect occur, (H.R > 1); increase in hazard, (H.R < 1): Reduction in hazard,

#### 2.2.5 Hypothesis

These are 12 hypotheses drawn from the diagnoses of COPD patients who have COVID‐19.

**H**_**1**_ = Fever has a significant effect on COPD.

**H**_**1**_ = Cough has a significant effect on COPD.

**H**_**1**_ = Sputum has a significant effect on COPD.

**H**_**1**_ = LRTI Infection has a significant effect on COPD.

**H**_**1**_ = AFB has a significant effect on COPD.

**H**_**1**_ = Normal condition has a significant effect on COPD.

**H**_**1**_ = SOB has a significant effect on COPD.

**H**_**1**_ = body‐aches has a significant effect on COPD.

**H**_**1**_ = Headache has a significant effect on COPD

**H**_**1**_ = Wheezing has a significant effect on COPD.

**H**_**1**_ = Right side chest pain has a significant effect on COPD.

**H**_**1**_ = TB/PTB has a significant effect on COPD.

## 3 RESULTS

In the descriptive analysis (Table 1), patients were categorized by gender, with males and females having 76.8% and 23.2%, respectively. Patients with COPD had an age range of 14–34, 34–54, 54–74, and 74–84 with 12.5%, 39.3%, 44.6%, and 3.6%, respectively (*p* = .000), and 82.9% survived during hospital treatment, while 17.1% died (*p* = .000). In district Muzaffar‐garh tehsil, namely, Muzaffar‐garh, Kot‐Adu, Jatoi, and Ali‐Pur, the patient ratio was distinguished with respect to 53.9%, 12.5%, 22.9%, and 10.7% of those treated with COPD that also have COVID‐19 disease. During the treatment, the patient had symptoms like fever, cough, sputum, LRTI, AFB, TB/PTB, normal condition, SOB, body‐aches, headache, wheezing, and right side chest pain with 54.6%, 87.1%, 42.5%, 9.3%, 36.4%, 43.6%, 17.1%, 92.1%, 65%, 96.4%, 9.3%, and 63.9%.

**Table 1:** Descriptive statistics of chronic obstructive pulmonary disease patients with COVID‐19 (*n* = 280).

Characteristics	Value (%)	*p*‐Value*
Gender		
Male	215 (76.8)	.047
Female	65 (23.2)	
Age group (y)		
14–34	35 (12.5)	.000
34–54	110 (39.3)	
54–74	125 (44.6)	
74–84	10 (3.6)	
Area (Tehsil‐wise patients)**		
Muzaffargarh	151 (53.9)	.901
Kot addu	35 (12.5)	
Jatoi	64 (22.9)	
Ali Pur	30 (10.7)	
Survival		
Live	232 (82.9)	.000
Death	48 (17.1)	.000
Symptoms		
Fever	153 (54.6)	.977
Cough	244 (87.1)	.000
Sputum	119 (42.5)	.000
LRTI	26 (9.3)	.000
AFB	102 (36.4)	.000
TB/PTB	122 (43.6)	.000
Normal condition	48 (17.1)	.000
SOB	258 (92.1)	.004
Body‐aches	182 (65)	.000
Headache	270 (96.4)	.020
Wheezing	26 (9.3)	.000
Right side chest pain	179 (63.9)	.000

Abbreviations: AFB, Acid Fast bacillus; COVID‐19, coronavirus disease 2019; LRTI, lower respiratory tract Infection; PTB, pulmonary tuberculosis; SOB, shortening of breath; TB, tuberculosis.

* *p*‐Value (<.05) is significant.

** Patients of subdistrict, namely, as Tehsil‐wise patients.

### 3.1 Model fitting information

**Figure 1:**
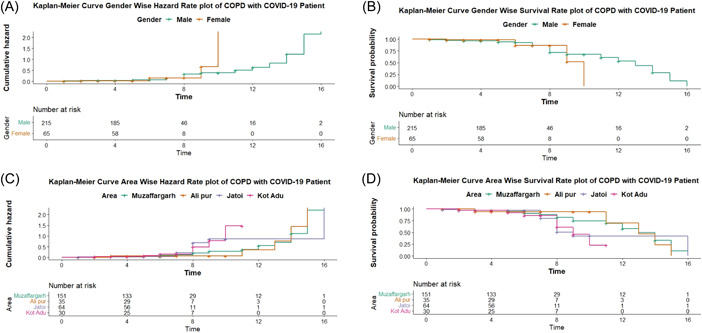
(A) Kaplan–Meier curve gender‐wise hazard rate plot of COPD with COVID‐19 patients. (B) Kaplan‐Meier curve gender‐wise survival rate plot of COPD with COVID‐19 patients. (C) Kaplan–Meier curve area‐wise hazard rate plot of COPD with COVID‐19 patients. (D) Kaplan–Meier curve area‐wise survival rate plot of COPD with COVID‐19 patients. COPD, COPD, chronic obstruct pulmonary disease; COVID‐19, coronavirus disease 2019.

In (Table 2), the Cox proportional hazard model likelihood of the Omnibus Test, the Chi‐square ratio represents the model's significance at (*p* = .051), and it demonstrates a strong association among independent variables. The graph's result pattern is depicted by Kaplan–Meier curves based on gender and area: (a) Time to Hazard gender‐wise graph (b) time to survival gender‐wise graph (c) area‐wise graph of time to hazards (d) area‐wise graph of time to survival. In (Figure 1A) and (Figure 1B), the hazards and survival figures rectify the average number of patients who survive or died over time. Gender‐wise graphs of hazards and survival corroborate that average females with COVID‐19 recover earlier than males. In the area‐wise graph, (Figure 1C,D), which reveal the patient survival in each subdistrict with respect to time, the survival time was observed to be approximately 10–16 days, and the death duration average 0–9 days for admitted patients.

**Table 2:** Cox proportional hazard model.

−2 Log likelihood	Overall (score)			Change from previous step			Change from previous block		
	Chi‐square	*df*	Sig.	Chi‐square	*df*	Sig.	Chi‐square	*df*	Sig.
355.593	20.932	12	.051	18.520	12	.101	18.520	12	.101

### 3.2 Negative binomial distribution representation

The negative binomial regression coefficients for each predictor variable, along with their standard errors, Wald chi‐square values, *p*‐values, and 95% confidence intervals, were reported as the parameter estimates as shown in (Table 3). Cough, LRTI, TB/PTB, normal condition, and body‐aches were categorized as significant dummy variables. This means that if one unit increases in these significant variables, the relative risk increases by 1.369 in cough, 0.693 in LRTI, 0.173 in TB/PTB, and 0.217 in body‐aches while decreasing by 0.396 in normal conditions. There appeared over‐dispersion in the estimate (variance greater than the mean); less‐than‐zero estimate suggests rare under‐dispersion.

**Table 3:** Parameter estimates of negative binomial regression.

Parameter	*β* a	Std. error	95% Wald confidence Interval		Hypothesis test			*EXP*(*β*)	95% Wald confidence Interval	
			Lower	Upper	Wald chi‐Square	*df*	Sig.		Lower	Upper
Intercept	0.460	0.3715	−0.268	1.188	1.536	1	.215	1.585	0.765	3.282
Fever	0.046	0.0645	−0.080	0.173	.517	1	.472	1.047	0.9231	1.188
Cough	1.369	0.3176	0.747	1.992	18.583	1	.000	3.932	2.110	7.329
Sputum	4.81E	0.2886	−0.566	0.566	0.000	1	1.000	1.000	0.568	1.761
LRTI	0.693	0.2360	0.231	1.156	8.624	1	.003	2.000	1.259	3.176
AFB	−0.140	0.2220	−0.575	0.295	.398	1	.528	0.869	0.563	1.343
TB/PTB	0.170	0.0826	0.008	0.332	4.250	1	.039	1.186	1.008	1.394
Normal condition	−0.396	0.0953	−0.583	−0.210	17.290	1	.000	0.673	0.558	0.811
SOB	−3.55	0.2499	−0.490	0.490	0.000	1	1.000	1.000	0.613	1.632
Body‐aches	0.217	0.1141	−0.007	0.440	3.608	1	.058	1.242	0.993	1.553
Headache	0.313	0.3123	−0.299	0.925	1.002	1	.317	1.367	0.741	2.521
Wheezing	0.169	0.1311	−0.088	0.426	1.665	1	.197	1.184	0.916	1.532
Right side chest pain	−0.098	0.1109	−0.316	0.119	0.786	1	.375	0.906	0.729	1.126
(Scale)	0.042	0.0073	0.030	0.059						

*Note*: Dependent variable: Age. Model: (intercept), fever, cough, sputum, LRTI, AFB, TB/PTB, normal condition, SOB, body‐aches, headache, wheezing, right side chest pain (a).

Abbreviations: AFB, Acid Fast bacillus; LRTI, lower respiratory tract Infection; PTB, pulmonary tuberculosis; SOB, shortening of breath; TB, tuberculosis.

^a^ Maximum likelihood estimate.

Likelihood of the Omnibus Test, Chi‐square ratio (Table 4) represents a model compared without any predictors (null hypothesis). The model is significant to intercept variables like cough, sputum, LRTI, AFB, TB/PTB, normal condition, SOB, body‐aches, headache, wheezing, and Right side chest pain that can change over time.

**Table 4:** Omnibus Test of negative binomial regression.

Likelihood ratio chi square^a^	*df*	Sig.
163.418	12	.000

*Note*: Dependent variable: Age. Model: (intercept), fever, cough, sputum, LRTI, AFB, TB/PTB, normal condition, SOB, body‐aches, headache, wheezing, right side chest pain (a).

Abbreviations: AFB, Acid Fast bacillus; LRTI, lower respiratory tract Infection; PTB, pulmonary tuberculosis; SOB, shortening of breath; TB, tuberculosis.

^a^ Compares the fitted model with the intercept‐only model.

## 4 DISCUSSION

The study examined a highly influential independent factor in COPD patients with COVID‐19.

The increased risk of death in COPD patients was due to the effects of variables such as cough, LRTI, TB, and body‐aches being 1.369, 0.693, 0.170, and 0.217 times higher at (95% CI: 0.747–1.992), (95% CI: 0.231–1.156), (95% CI: 0.008–0.332), and (95% CI: −0.07 to 0.440) while it decreased 0.396 in normal condition. Patients diagnosed with COPD had a higher mortality rate and showed less improvement over time. Furthermore, our findings demonstrated that COVID‐19 case fatality rates were higher in regions with a higher prevalence of COPD. Similarly, the number of cases of COVID‐19 is increasing exponentially in many developing countries where rates of COPD are rising and there is a notable disparity between illnesses.^32^ To mitigate the effects of COVID‐19, fundamental strategies for people with COPD are needed. According to the WHO report of December 12, 2022,^33^ there had been 651,918,402 confirmed cases of COVID‐19 and 6,459,018 deaths reported.

A larger sample size is desirable, but multiple factors affect study conclusions. Our 280‐patient study draws meaningful and useful conclusions by ensuring sample representativeness, using appropriate statistical techniques, considering effect size and precision, relating findings to existing evidence, and acknowledging limitations. From a total of 280 patients, 76.8% were males and 23.2% were females; we divided them into groups at suitable intervals between the ages of 14 and 84. The Cox proportional hazard model revealed a strong association between the variables, and the negative binomial distribution indicates that the model was significant, as well as the patients' relative risk. Results confirmed that the survival rate decreased when the patient was in a critical situation. The treatment was highly effective for COPD patients who had cough, LRTI, TB/PTB, and bodyaches. Several studies have examined at the correlation between SARS‐CoV‐2 and COVID‐19 status; individuals with COPD are more likely to worsen if they test positive. In the related cohort study, symptoms such as confusion, anxiety, weariness, exertional dyspnea, sleeplessness, sadness, cough, and bowel problems were the most prevalent with (prevalence > 5%) symptoms at 12‐month contact.^34^ COPD patients who received COVID‐19 had a significantly worse disease prognosis as measured by hospitalizations (31.1% vs. 39.8%: OR 1.57; 95% CI 1.14–1.18) and mortality (3.4% vs. 9.3%: OR 2.93; 95% CI 2.27–3.79).^35^ Another researcher reported that people with COPD appear to have a modestly increased risk of severe disease, and the risk of death from COVID‐19 at the height of the epidemic was mostly lower.^36^ This happens a lot in North America and Europe, where healthcare professionals and public health workers from the GOLD (Global Initiative for Chronic Obstructive Lung Disease) study a group of COPD patients without any bias. Symptoms found that dyspnea, wheezing, chest infection, sputum production, weight loss, and cough that led to poor health.^26^

Our research shows that COPD patients' risk of dying from any cause is affected more by their diagnosis than by demographic variables like age, gender, or location. However, owing to a lack of patients with severe COPD, we were unable to accurately assess the effect of severity, therefore, these findings should be interpreted with caution. Despite our best efforts, we may have overlooked mild instances of COPD when no medicine was used for maintenance. Having individuals with COPD and no other diseases would have made it simpler to rule out other causes, but we missed this information in our research, so our estimations could be inaccurate. We excluded persons whose dates were missing from the survival analysis. To examine the dispersion of potential confounding variables in the samples used for each time‐to‐event study, statistical tests were performed. It made sure that COPD patients weren't drastically different from one another. The key outcomes of death and recovery may be lacking some data. This was remedied by excluding patients who did not reach a final result on the last day of follow‐up from the survival analysis (LAMA cases).

This may have affected the comparison between those with severe and those with mild COPD.^8^ Longitudinal investigations with larger samples are required to validate our findings, but to our knowledge, this is the first major research to examine the possible association between COPD and a high‐burden COVID‐19.

Furthermore, COVID‐19 and COPD are linked directly, while SARS‐CoV‐2 indirectly leads to COPD morbidity and death. Health systems worldwide were affected by COVID‐19, and it is predicted that medicine shortages and delays in the identification and treatment of COPD will increase death rates for persons with the disease.^37^, ^38^, ^39^ In addition, those already at a financial disadvantage are further impacted by rising medical expenses because of the unaffordability of the healthcare system. Our results demonstrate the need to maintain COPD routines and testing services as a top priority, even if the COVID‐19 pandemic has prompted adjustments to health and social systems.^40^, ^41^ When examining the role of COVID‐19 risk factors in patients with COPD, several factors can influence the severity and outcomes of COVID‐19 infections in this specific population. Age, smoking history, underlying lung function, co‐existing medical conditions, immune function, medication adherence to COPD treatment plans, vaccination against COVID‐19, and adherence to general preventive measures such as mask‐wearing, hand hygiene, and social distancing are necessory.^42^

Our results highlight the need of maintaining high standards for COPD screening and monitoring programs. To defeat the epidemic, strategic health planning and resource allocation were required. More extensive follow‐up research with large enough samples to draw meaningful conclusions is needed. This research contains enough patients, in light of what we found, to examine the relationship between COPD and a high‐burden lifestyle.

## 5 CONCLUSION

After scrutinizing 12 independent variables (diagnosis), this study reveals that cough; LRTI, TB/PTB, and bodyaches were the main causes of COPD in COVID‐19. Controlling these four factors reduces the chances of COPD patients contracting (COVID‐19 and SARS‐CoV‐2) and improves their chances of recovery. Our research has important implications for the organization of health care for COVID‐19 patients and for prioritizing health interventions and protection measures toward the most vulnerable chronic patients. Disease combinations should be evaluated for successful infection prevention in severe instances of infection.

## AUTHOR CONTRIBUTIONS

**Muhammad Muneeb Hassan**: Conceptualization; data curation; formal analysis; investigation; software; validation; visualization; writing—original draft; writing—review & editing. **M H Tahir**: Formal analysis; investigation; project administration; supervision; validation; visualization; writing—review & editing. **Muhammad Ameeq**: Conceptualization; data curation; formal analysis; investigation; methodology; resources; software; validation; visualization; writing—original draft; writing—review & editing. **Farrukh Jamal**: Formal analysis; investigation; methodology; project administration; supervision; visualization; writing—review & editing. **John T Mendy**: Formal analysis; project administration; supervision; validation; visualization; writing—original draft; writing—review & editing. **Christophe Chesneau**: Investigation; writing—original draft; writing—review & editing.

## CONFLICT OF INTEREST STATEMENT

The authors declare no conflict of interest.

## ETHICS STATEMENT

The review board statement of this study is available from the corresponding author upon reasonable request.

## INFORMED CONSENT STATEMENT

Not applicable, because secondary data of discharged patients were collected from the hospital record with the approval of the ethical committee.

## Data Availability

The DHQ Hospital Muzaffar‐garh Statistical Officer in charge of ethics committee provided a letter of clearance for the study on May 10, 2022, citing the number 1174‐77/DHQ. All data, models, or code that supports the findings of this study is available from the corresponding author upon reasonable request. All data, models, or code that supports the findings of this study is available from the corresponding author upon reasonable request.
